# Understanding the Context for Incorporating Equity into Quality Improvement Throughout a National Health Care System

**DOI:** 10.1089/heq.2023.0009

**Published:** 2023-05-26

**Authors:** Leslie R.M. Hausmann, Carolyn Lamorte, Jamie L. Estock

**Affiliations:** ^1^ Center for Health Equity Research and Promotion (CHERP), Veterans Affairs (VA) Pittsburgh Healthcare System, Pittsburgh, Pennsylvania, USA.; ^2^ Division of General Internal Medicine, Department of Medicine, University of Pittsburgh, Pittsburgh, Pennsylvania, USA.

**Keywords:** health care equity, veterans, quality improvement, user-centered design

## Abstract

**Purpose:**

Although health care systems aspire to deliver equitable care, practical tools that empower the health care workforce to weave equity throughout quality improvement (QI) processes are lacking. In this article, we report findings from context of use interviews that informed the development of a user-centered tool to support equity-focused QI.

**Methods:**

Semistructured interviews were conducted from February to April of 2019. Participants included 14 medical center administrators, departmental or service line leaders, and clinical staff involved in direct patient care from three Veterans Affairs (VA) Medical Centers within a single region. Interviews covered existing practices for monitoring health care quality (i.e., priorities, tasks, workflow, and resources) and explored how equity data might fit into current processes. Themes extracted through rapid qualitative analysis were used to draft initial functional requirements for a tool to support equity-focused QI.

**Results:**

Although the potential value of examining disparities in health care quality was clearly recognized, the data necessary for examining disparities were lacking for most quality measures. Interviewees also desired guidance on how inequities could be addressed through QI. The ways in which QI initiatives were selected, carried out, and supported also had important design implications for tools to support equity-focused QI.

**Discussion:**

The themes identified in this work guided the development of a national VA Primary Care Equity Dashboard to support equity-focused QI within VA. Understanding the ways in which QI was carried out across multiple levels of the organization provided a successful foundation upon which to build functional tools to support thoughtful engagement around equity in clinical settings.

## Introduction

The Veterans Health Administration within the Department of Veterans Affairs (VA) has long recognized the importance of health equity through its strong support of health services research to advance health and health care equity among Veterans^1–11^ and creation of the national Office of Health Equity.^12^ Establishment of national centers and programs focused on at-risk or marginalized populations, including Veterans experiencing homelessness, women, racial and ethnic minority Veterans, and rural-dwelling Veterans, among others, further demonstrates VA's commitment to serving the needs of all Veterans.^13–19^

Although VA has been a leader in making health equity a priority, the Veterans Chartbook released by the Agency for Healthcare Research and Quality in 2020 showed that female Veterans and Veterans from minoritized racial and ethnic groups continue to have worse access to health care and higher mortality rates than their male and non-Hispanic white counterparts.^20^ Persistent inequities such as these underscore the need for new tools for pursuing equity in VA health care settings.

There is no shortage of blueprints, roadmaps, and calls to action that provide guidance on how to address inequities from within the health care system.^21–27^ Much of this guidance focuses on incorporating equity into the quality monitoring and improvement processes that guide organizational priorities and clinical operations.^21,28–32^ The first step in this process is often to examine quality data stratified by race/ethnicity, gender, and other patient demographic characteristics. Quality monitoring tools (e.g., dashboards) designed to highlight disparities in quality metrics have started to emerge.^33,34^

While these dashboards reflect health care systems' recognition and commitment to reducing health care disparities, they are only a first step toward fully integrating equity into quality improvement (QI) initiatives.^21,33,34^ Practical tools that empower QI teams to weave equity throughout their work have yet to be developed, and current quality monitoring practices continue to lack an explicit focus on equity.^35,36^ To facilitate a true paradigm shift in the way QI is conducted, it is necessary to understand the current workflow and environmental constraints in which QI teams operate, and identify multiple points in the QI process when equity should be considered.

Toward this end, we sought to understand the context for integrating equity into the current VA QI process. This work was the foundation for a broader initiative to develop a multifaceted user-centered tool that would both highlight health care inequities within VA Medical Centers (VAMCs) and empower members of the health care workforce to approach QI through an equity lens.

## Methods

### Overview

In this article, we focus on the first stage of a user-centered design (UCD) process to support equity-focused QI in VA. UCD aims to create tools that are usable and useful by applying human factor knowledge and usability methods to improve the likelihood of adoption and sustained use.^37^ UCD places end users at the center of an iterative process that seeks to understand the responsibilities and requirements of users before designing the tool rather than designing the tool and subsequently trying to obtain user buy-in.^38^

**FIG. 1.**
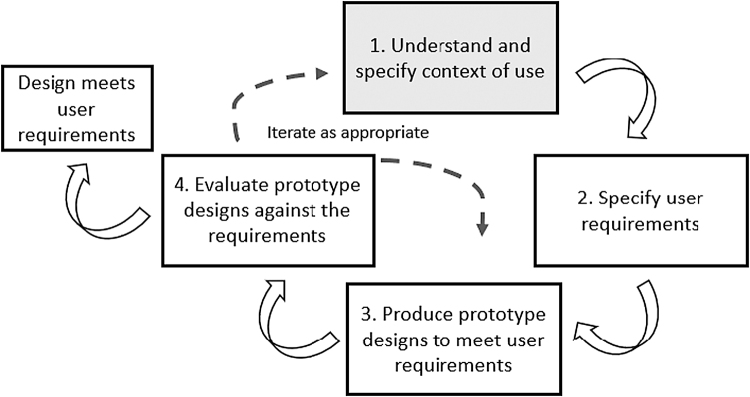
Overview of UCD process. Adapted from ISO (2019).^37^

The UCD process starts with seeking an in-depth understanding of the realities and constraints of the context in which end users will interact with the tool, such as their priorities, tasks, workflow, and resources (Fig. 1). The next step is specifying user and organizational requirements, which are then used to produce simple design solutions, or prototypes. The prototypes are then evaluated and refined until they meet all user requirements. In this article, we report on themes identified from context of use interviews, as they illuminated important design considerations for tools to support incorporating equity into the QI process. This work was recognized as QI and deemed exempt from human subjects review by the Institutional Review Board at the VA Pittsburgh Healthcare System. We followed the Consolidated criteria for REporting Qualitative (COREQ) research in describing this work.^39^

### Participants and recruitment

Our population of interest included members of the VA health care workforce who are directly involved in prioritizing, directing, or conducting QI initiatives in VA primary care settings in one regional VA network. To understand how priorities are set at multiple levels of the organization, we interviewed medical center administrators, departmental or service line leaders, and clinical staff involved in direct patient care. All interviewees were in roles where they interacted with QI data in some capacity. Interviewees were identified using purposeful sampling^40^ and were contacted by interviewers by email. Most interviewees were familiar with the interviewers through their shared place of employment.

### Data collection and analysis

Semistructured interviews were conducted in-person or by telephone, with email follow-up for clarification or additional questions as needed. Interviews lasted ∼30 min and were conducted from February to April of 2019. Interviewers included the three co-authors, working alone or in pairs. All interviewers were experienced in conducting semistructured interviews and were trained in the social sciences (C.L., social worker; J.L.E., human factors psychologist; and L.R.M.H., social psychologist and health equity researcher). Most interviewees were interviewed individually apart from one instance in which two people were interviewed together.

Interviews were designed to understand existing practices for monitoring health care quality (i.e., priorities, tasks, workflow, and resources) and explore how equity data might fit into current QI processes. Interviewees were asked to identify high-priority quality measures, describe who was involved in tracking quality data and how measures were tracked, and report whether disparities by race and ethnicity, gender, or geography were being monitored. Questions also asked about past or ongoing QI initiatives, whether such initiatives were explicitly focused on disparities, and how information about disparities, if made available, would likely be utilized in future QI efforts.

Themes were extracted through rapid qualitative analysis.^41^ Interviewers summarized key points from each interview into an excel spreadsheet. Interviewers then reviewed the summaries after every two to three interviews and identified areas to probe further in subsequent interviews. The process continued until saturation was reached.^42^ Interview summaries were then used to draft an initial set of interface design and functional requirements for developing a tool to support equity-focused QI.

## Results

### Sample characteristics

We conducted a total of 13 interview with 14 respondents (36% female) from three VA medical facilities of varying complexity within a single region (Table 1). Interviewees occupied roles that involved medical center leadership (e.g., chief of staff, service line chief) and direct patient care (e.g., physician, nurse, pharmacist) and had varying levels of responsibility for patient outcomes on targeted measures of chronic disease management.

**Table 1.** Professional Roles of Interviewees Drawn from Three Veterans Affairs Medical Centers of Varying Complexity Within a Single Region

Professional role	Number of interviewees
Chief of staff	3
Associate chief nurse	1
Quality performance specialist	1
Service line chief, primary care and behavioral health	2
Clinical nurse specialist, primary care	1
Nurse manager, primary care	1
Physician, primary care	4
Pharmacist, primary care	1
Total	14

### Key insights

Interviewees described typical processes for planning and carrying out QI, including how priorities are set, who is involved, how data are used to inform interventions and track progress, if/how equity is incorporated into current workflow, and what resources exist to support initiatives. Below we summarize insights that emerged from these interviews, organized into themes related to QI priorities, tasks and workflows, and resources.

### Priorities

#### VA QI priorities are both identified from the top-down and emerge from the bottom-up

Given the VA's hierarchical structure, most strategic priorities discussed by interviewees were top-down in nature. Priorities set by VA Central Office become national priorities, which influence regional network annual plans, which in turn influence priorities and QI goals at individual facilities. Interviewees conveyed that the formula used to rank individual VA facilities in terms of quality (i.e., VA's Strategic Analytics for Improvement and Learning Value Model, or SAIL) prompted local leaders to strive for overall improvement relative to other VAMCs. Described by one chief of staff, “We are looking to be at the top of quality indicators. It is a ranked system, so you want to achieve the highest ranking possible.”

Acknowledging the influence of national quality priorities, respondents noted that priorities also emerged from the bottom-up based on local needs. Another chief of staff described planning for “reactive” initiatives in response to things that go wrong, while many department leaders and front-line providers reported identifying areas for improvement based on trends observed in the local patient population.

#### The potential value of examining disparities in health care quality is clearly recognized

Even though disparities were not routinely examined or prioritized in practice, respondents generally acknowledged that stratifying quality measures by demographic groups could be useful. One provider felt that having race, gender, and regional information about patients could be helpful in targeting efforts to reduce readmission rates for ambulatory sensitive conditions. A primary care chief shared that examining quality measures by race could help raise provider awareness about things like racial bias. A chief of staff remarked, “I would love to see all demographic data for Veterans be integrated into all data registries to be used by folks in innovative ways.” They further acknowledged that where people live and how much money people make, characteristics that are not widely available in existing VA data sources, have a huge influence on health.

### Tasks and workflow

#### Improvement priorities are based on aggregated data, while more granular data are used to investigate and intervene on quality issues

Data are needed at multiple levels of aggregation throughout the QI process. Medical center administrators rely on aggregated data when reviewing quality at the facility level and setting improvement priorities. As potential issues are flagged, data are examined at more granular levels to pinpoint specific clinical settings in which quality lags.

The concept of “drilling down” from highly aggregated reports to team- and patient-level data was a common theme across interviewees. One primary care chief detailed a typical process of presenting aggregated data to providers followed by generating and distributing provider or team-specific reports of patients who were not meeting the measure. The purpose of drilling down to “patient outlier” reports was at least threefold: investigate why patients may not be meeting the quality measure, inform clinical interventions, and support proactive outreach.

#### Implementation of QI is often delegated to individual service lines, clinical teams, and providers

Regardless of the level at which priorities are set or quality issues are identified, implementing improvement practices is typically delegated to members of the clinical workforce. When medical center leadership prioritized quality measures, for example, it was common practice to task frontline staff members in the form of committees, workgroups, or individuals (e.g., nurses, pharmacists, or residents) with reviewing data and carrying out improvement strategies. An associate chief nurse noted that high-priority performance measures were “funneled down to nurse managers and nurses in each unit.” Those nurses then conducted locally tailored projects to investigate and address root causes of quality issues within their clinical settings.

#### The tasks and responsibilities of direct patient care often leave providers little time for systemic QI efforts

Even though those involved in direct patient care were tapped to assist with addressing quality issues, providers without a designated QI role expressed that they lack adequate time to do anything other than see patients. When asked about the extent to which quality measures are used in their own practice, one physician answered, “Not much. We are overwhelmed with data. We need protected time to do anything other than clinical work.” Another described, “I don't use tools directly because I don't have bandwidth to do it. Everyone agrees it's something we should do, but you've got to find the best way to work it into the process.”

### Resources

#### The data necessary for examining disparities are lacking for most quality measures

Interviewees explained that examining disparities in quality measures was rare. Most respondents (12 of 14) said that they do not routinely see facility or service line-level quality reports stratified by race and ethnicity, gender, geographic location, or other disparity-related variables. Although some respondents had seen reports stratified by race, such reports were not standard. One chief of staff explained, “Measures are not usually broken down that way. You may be able to drill down to those factors, but I am not sure how easy it would be.”

Demographic characteristics were also rarely and inconsistently included in patient-level reports. Some interviewees shared that binary gender, geographic region (rural vs. urban residence), and age were sometimes included, although this was highly dependent on the data source. Race and ethnicity were nearly always omitted from patient-level reports. Instances in which race and ethnicity had been included were in reference to a past QI initiative that explicitly focused on addressing racial disparities in hypertension.^3^

#### Clear and actionable guidance should be provided on how inequities could be addressed through QI

Interviewees widely agreed that they would benefit from expert recommendations on interpreting equity data, designing equity-focused projects, and using evidence-based interventions to reduce disparities. Generic or universal improvement strategies will be the default without support from health equity experts. When referring to an example of an improvement initiative inspired by data showing that blood pressure control targets were lower for Black Veterans, a respondent shared that, “We targeted our QI effort toward the entire population of outliers because we felt these interventions could help everyone.” QI teams may opt for generic or universally applied approaches that are seen as valuable due to their feasibility or broad reach, with the assumption that minoritized patient groups will see the same benefit from these interventions as the majority.

## Discussion

This UCD-driven, qualitative inquiry into current VA QI practices provided several critical insights regarding what will be needed to fully weave equity into the QI culture within the VA and the context in which a health equity-focused QI tool would be used. The themes we identified have several implications for designing tools that make equity a core consideration throughout all stages of QI.

First, equity needs to be incorporated into quality monitoring practices at the national level, given that what is prioritized in national quality monitoring practices heavily influences regional and local priorities. Because the VA's current quality ranking system does not include equity as an explicit element, regional and local efforts to improve quality rankings rarely focus explicitly on reducing inequities as opposed to elevating quality overall. For equity to be fully integrated throughout VA's QI culture, methods that monitor and rank facilities in terms of quality need to incorporate measures of variability in performance across historically marginalized or minoritized Veteran populations.^33,43^

Including indicators that convey how consistently each facility delivers high-quality care regardless of patient demographic characteristics would not only demonstrate VA's commitment to equity as one of the pillars of health care quality but would also provide facilities with equity-specific targets to strive toward when considering local QI priorities. Applying analytic methods that decompose disparities into within and between facility components could further inform efforts to identify the largest and most persistent disparities affecting the VA patient populations.^43–47^ Applying such methods at a national level would also provide guidance to VA in terms of which facilities to engage in equity-focused improvement initiatives and how to prioritize patients from marginalized populations to close quality gaps.

Second, building equity into multiple levels of infrastructure will be needed to keep equity top of mind throughout the QI process. National, regional, and local decision support tools need to include the capability to stratify and compare quality measures by demographic groups defined by race/ethnicity, gender, and other proxy markers for historical, social, and economic disadvantage. Disaggregating data in regional and local quality reporting tools would facilitate the consideration of equity-related issues when developing regional and local priorities and performance plans. Given that responsibility for exploring causes of quality issues and carrying out improvement initiatives is often delegated to local people or teams within specific clinical areas, such teams also need to be equipped with the data and protected time required to investigate and intervene on root causes of inequities.

Demographic characteristics and indicators of social determinants of health that are available in the electronic health record should be included in all patient-level quality reports. The inclusion of this information would facilitate in-depth exploration into potential root causes of quality issues that may be disproportionately affecting the health care of certain patient subgroups versus those that are common across all patients. Including patient demographic information would also facilitate efficient outreach to populations that are experiencing disparate care and may need tailored improvement interventions.

Third, the practice of delegating responsibility for improving quality to individual teams and providers poses potential barriers to incorporating equity into the QI process. For example, a common scenario is that a quality gap is identified at a facility level, and then teams and providers are called upon to help improve that quality measure for their individual patients. They may be provided with clinical reminders or lists of patients who have not received the recommended level of care, and then tasked with addressing the quality issue for their patients. Our findings showed that providers involved in direct care of patients often do not have the protected time to investigate or address quality issues at a population level.

Furthermore, disparities are system-level issues caused by multiple factors, many of which are outside the scope of what individual providers or teams can address within their regular work. Considering system-level policies, practices, and potential biases as possible root causes of disparities should be part of the conversation when designing equity-focused improvement initiatives. The dominant model of delegating the responsibility for improving quality to individual teams and providers is not conducive to engaging the workforce in the collective action and system changes needed to address disparities in quality.

Finally, systems for providing equity training and resources will be essential to advancing equity-focused improvement initiatives. Training and support should emphasize the bigger goals of the QI initiative and how individuals can contribute to positive change. To foster a sense of urgency to act on disparities within our personal spheres of influence, members of the health care workforce should be given opportunities to learn about social and structural determinants of health equity and the ways in which health care policies have caused and perpetuate inequities.^48–52^ To demonstrate that it is possible to reduce disparities when equity is an explicit consideration of QI, members of the health care workforce should also be trained on promising practices for reducing disparities.

Such promising practices include developing interventions with multiple targets for change (e.g., patients, providers, system), leveraging community health workers or peers, culturally tailoring interventions to the needs and preferences of marginalized groups, and empowering nurses to lead QI initiatives.^53^ Teams should also be encouraged to partner with clinical staff who work with marginalized Veterans and engage Veterans from marginalized groups when designing equity-focused QI to assure that new initiatives benefit the target group, are designed to be sustained and spread, and do not have unintended negative consequences.^54–56^ Making publicly available toolkits, tailored patient educational materials, and other equity-oriented resources easily accessible could also help jump start new equity-focused improvement initiatives.

### Application of knowledge gained

The themes identified in this work guided our initial development of tools and processes to support equity-focused QI within VA. Understanding the overall ways in which QI was carried out across multiple levels of the organization helped us to distinguish two categories of users with differing needs: (1) decision-makers who set QI priorities and (2) frontline staff who design, implement, and evaluate interventions. We chose to focus initially on the second set of users, given that this group plays an essential role in the success of many improvement initiatives, yet expressed not having adequate time or infrastructure to support QI activities.

Continuing with the steps of the UCD process,^37^ we developed a national VA Primary Care Equity Dashboard (PCED) to meet the needs of QI champions working in VA Primary Care settings as they plan, design, implement, and evaluate equity-focused QI projects in their local VAMCs. Hosted on the VHA Office of Health Equity SharePoint site, the PCED contains a series of reports that show both quality and equity in select outpatient measures tracked by the VA Office of Reporting, Analytics, Performance, Improvement, and Deployment.^57^

Importantly, the PCED shows how individual VAMCs compare to the national average on each measure overall and by subgroups defined by racial/ethnic group, gender/sex, rural/urban residence, and neighborhood poverty level. The PCED also includes patient-level reports that contain patient demographic variables as well as clinical information about patients who have not yet received the recommended level of quality for a particular measure. To assist end users with planning initiatives, the PCED also contains a curated library of relevant evidence-based equity-focused resources, and additional guidance on how to use the PCED is available on the PCED SharePoint Site.

First released on a national scale in February 2021,^58^ the PCED now has over 1600 users spanning all 23 Veterans Integrated Service Networks and VA Central Office. Case studies based on the PCED have been featured in a course sponsored by VHA Office of Patient Centered Care and Cultural Transformation on social and structural determinants of health, and projects inspired and supported by the PCED have been presented as Health Equity Safety stories to the VA Governance Board. Based on the initial uptake of the PCED and early success stories, the PCED provides a successful model for how to build functional tools that support thoughtful engagement around equity in clinical settings.

## Abbreviations Used

COREQ: Consolidated criteria for REporting Qualitative
EQuAl: Equity and Quality Aligned
PCED: Primary Care Equity Dashboard
QI: quality improvement
UCD: user-centered design
VA: Veterans Affairs
VAMCs: VA Medical Centers
